# Serum parameters and energy balance during the Yukon Arctic Ultra: a multistage ultramarathon in Arctic conditions

**DOI:** 10.3389/fnut.2025.1691771

**Published:** 2026-01-20

**Authors:** Adriane K. Schalt, Robert H. Coker, Hanns-Christian Gunga, Camilla Kienast, Lea Mascarell-Maricic, Mathias Steinach

**Affiliations:** 1 Center for Space Medicine and Extreme Environments Berlin, Institute of Physiology, Charité – Universitätsmedizin Berlin, Corporate Member of Freie Universität Berlin, Humboldt-Universität zu Berlin, Berlin, Germany; 2 Montana Center for Work Physiology and Exercise Metabolism, University of Montana, Missoula, MT, United States; 3 Charité – Universitätsmedizin Berlin, Corporate Member of Freie Universität Berlin, Humboldt-Universität zu Berlin, Clinic for Psychiatry and Psychotherapy, Berlin, Germany

**Keywords:** blood analysis, cold, endurance, energy balance, energy expenditure, serum parameter, subarctic, ultramarathon

## Abstract

**Purpose:**

The objective of this study is to focus on energy intake and expenditure, as well as changes in various serum parameters regarding stress and metabolism, during the Yukon Arctic Ultra (YAU), an ultramarathon of 690 km length, under Arctic conditions.

**Methods:**

The Yukon Arctic Ultra was studied over 4 years (2013, 2015, 2017, and 2019). A total of 22 participants (14 men, 8 women) were recruited, who raced on foot while pulling a sled. At four different checkpoints (PRE 0 km; D1 277 km; D2 383 km, and POST 690 km), measurements were performed.

**Results:**

A total of 14 participants finished (FIN) the race successfully (9 men; 5 women), 8 participants dropped out; total body weight loss in FIN men was 4.9 ± 2.1 kg, and in FIN women, 3.2 ± 1.8 kg. Total energy intake in FIN was 53,049 ± 10,474 kcal and 4,840 ± 819 kcal/day. Energy expenditure in FIN totalled 71,539 ± 10,585 kcal and 6,628 ± 1,019 kcal/day, resulting in a caloric deficit. Troponin T and CK showed significantly lower values in the slower participants at POST (diff means: 8.2 pg./mL and 417 U/L, respectively). CRP and NT-pro BNP increased at D1 (diff means: 16.5 mg/dL and 322.4 pg./mL, respectively), LDL decreased at POST (−45.5 mg/dL), as well as cholesterol, triglycerides, and non-HDL. Triiodothyronine (fT3) and thyroxine (fT4) decreased at POST (−1.0 and −1.8 pg./mL, respectively).

**Conclusion:**

A considerable energy deficit was identified in almost all athletes, which resulted in the loss of both lean mass and fat mass. Serum cardiac, lipid, and inflammation markers were altered significantly, indicating severe stress. It remains to be seen whether such events contribute to pathological sequelae or are merely temporary without clinical relevance.

## Highlights

- Energy balance and blood parameter changes in ultramarathon runners covering a 690 km distance under Arctic conditions
- Loss of body mass in FIN men: 4.9 kg and FIN women: 3.2 kg; total energy intake: 4,840 kcal/day; total energy expenditure: 6,628 kcal/day
- Favorable changes in lipid markers, increases in cardiac and inflammation markers, as well as decreases in thyroid hormones

## Introduction

Endurance sport is considered beneficial for the physical and mental health of humans (1, 2). Ultramarathon running refers to any distance exceeding 42.195 km, with the length, difficulty, and complexity of races varying greatly. Races take place under extreme conditions: for example, in terms of distance, the “Trans Europe Foot Race” covers 4,486 km (3); in terms of altitude, the “Tor de Geants” includes 24,000 m of ascent (4); in heat, races can reach temperatures up to 55 °C, e.g., “Marathon des Sables” (5); and in extreme cold, the “Yukon Arctic Ultra” (YAU) experiences temperatures below −40 °C (6).

However, it has been shown that beyond a certain duration threshold, the effects on an athlete’s health can become detrimental, with a higher incidence of injuries and increased inflammation (7–9). Nevertheless, the popularity of ultralong endurance events and sport in extreme environments has grown remarkably in recent years (10), alongside rising scientific interest. A great deal of attention is paid to nutrition to optimize performance (11). Athletes strive to match their energy expenditure, yet several previous studies have clearly pointed out that this is rarely achieved (12, 13), which consequently leads to a loss of muscle mass (14).

In addition, significant alterations in blood parameters have been observed during strenuous ultradistance running. These include changes in NT-pro BNP and Troponin T (15); increases in inflammation markers such as glutamate oxalacetate transaminase (GOT), glutamate pyruvate transaminase (GPT) (16), and creatine kinase (CK) increased (17, 18); and decreases in lipids, including triglycerides, high-density lipoprotein (HDL), low-density lipoprotein (LDL), and cholesterol (19, 20). Our previously published data from the same event also indicated changes in body mass and fat mass (FM), a decrease in parasympathetic drive, and changes in several metabolites, such as follistatin and irisin (6, 21, 22).

The focus of this paper is on energy intake and expenditure, as well as changes in selected serum parameters during the Yukon Arctic Ultra, a 690 km ultramarathon conducted under extremely cold conditions. The aim was to evaluate the degree to which they alter and whether these changes differ across specific subgroups, such as sex, age, BMI, speed, or distance covered.

## Materials and methods

### Trail and environment

**Figure 1:**
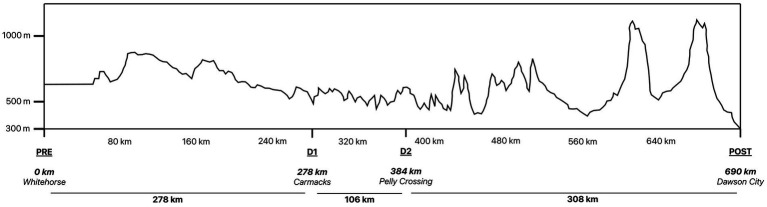
Route profile—adopted from “trackleaders.com” (95).

The Yukon Arctic Ultra is an ultramarathon that takes place biennially each February in the Yukon Territory. To increase the sample size, analyses were carried out across 4 different years (2013, 2015, 2017, and 2019). Participants aimed to finish a distance of 690 km, a trail along the Yukon River, with altitude variations of approximately 1,000 m. Temperature data were acquired from official weather reports published daily online by the Climate Services Office of Canada (23). Fatigue, hypothermia, and frostbite are common risks, contributing to an overall dropout rate of 61% between 2013 and 2019 (24). Survival training before the race is mandatory to ensure participants can demonstrate essential outdoor skills. All race equipment—such as food, clothing, a sleeping bag, a tent, and a GPS tracker—is stored on a pulka-sled (weight approximately 15–20 kg), which athletes pull throughout the race. Competitors can choose from three disciplines, walking, skiing, or mountain biking, over various distances. This analysis covers only the 690 km walking discipline Figure 1.

### Checkpoints

Ten different checkpoints had to be passed where athletes could rest and sleep. Pre-rationed meals were provided. Checkpoints differed in terms of facilities, e.g., an unheated barn, a farm, or a community center. Trained medical staff evaluated the athletes’ condition and, if deemed medically necessary, e.g., in cases of frostbite, removed competitors from the race. The distance between checkpoints ranged from 45 to 160 km.

**Figure 2:**
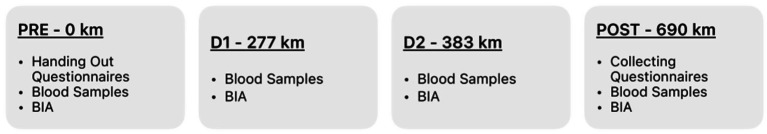
Research steps at check points.

At four checkpoints, PRE (0 km; Race Start; Whitehorse), D1 (277 km; Carmacks), D2 (383 km; Pelly Crossing), and POST (690 km; Finish; Dawson City), examinations were carried out and data were collected for the research study (Figure 2).

### Participants

A total of 22 healthy adult competitors, 14 men (M) and 8 women (W), took part in this study. All of them had experience in marathon running and ultraendurance racing; however, none were professional athletes. All adults taking part in the YAU were eligible for the study—there were no further inclusion criteria. Participants taking part in the study did not receive a financial incentive. The criteria for non-finishers (NON) to be included in the study were reaching at least D1 but not POST. Participants stated they had not used performance-enhancing drugs throughout the race. The study was approved by the Charité ethics board (review number EA/109/12), and all measurements and procedures complied with the Declaration of Helsinki (7th revised version, 64th World Medical Association meeting, Fortaleza, Brazil) regarding the treatment of human subjects.

### Subgroups

**Figure 3:**
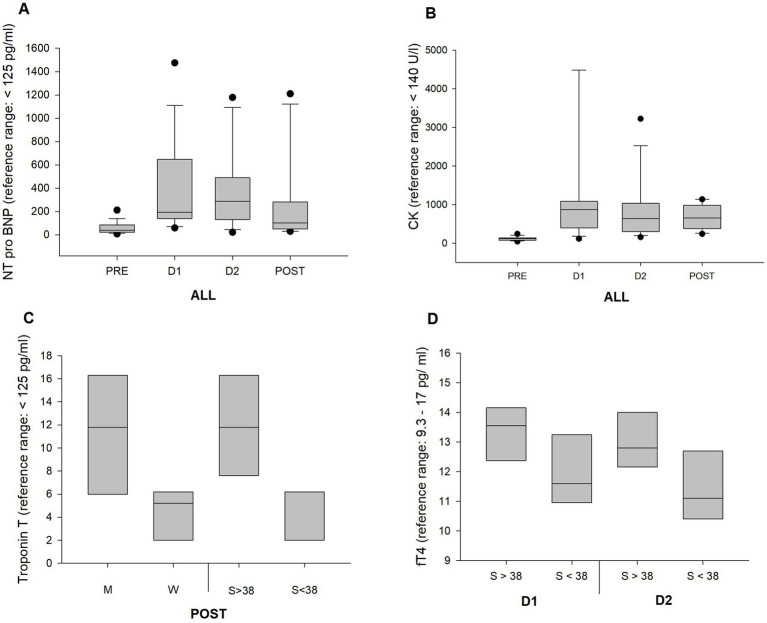
Boxplots represent changes in blood parameters over distance and within subgroups. **(A)** The change in NT-pro BNP with significant changes for PRE vs. D1 and D2. **(B)** CK-MB with a significant change at D1 vs. PRE and **(C)** for Troponin T at FIN for men vs. women, and faster vs. slower racers. **(D)** A higher fT4 in faster racers at D1, as well as at D2.

**Figure 4:**
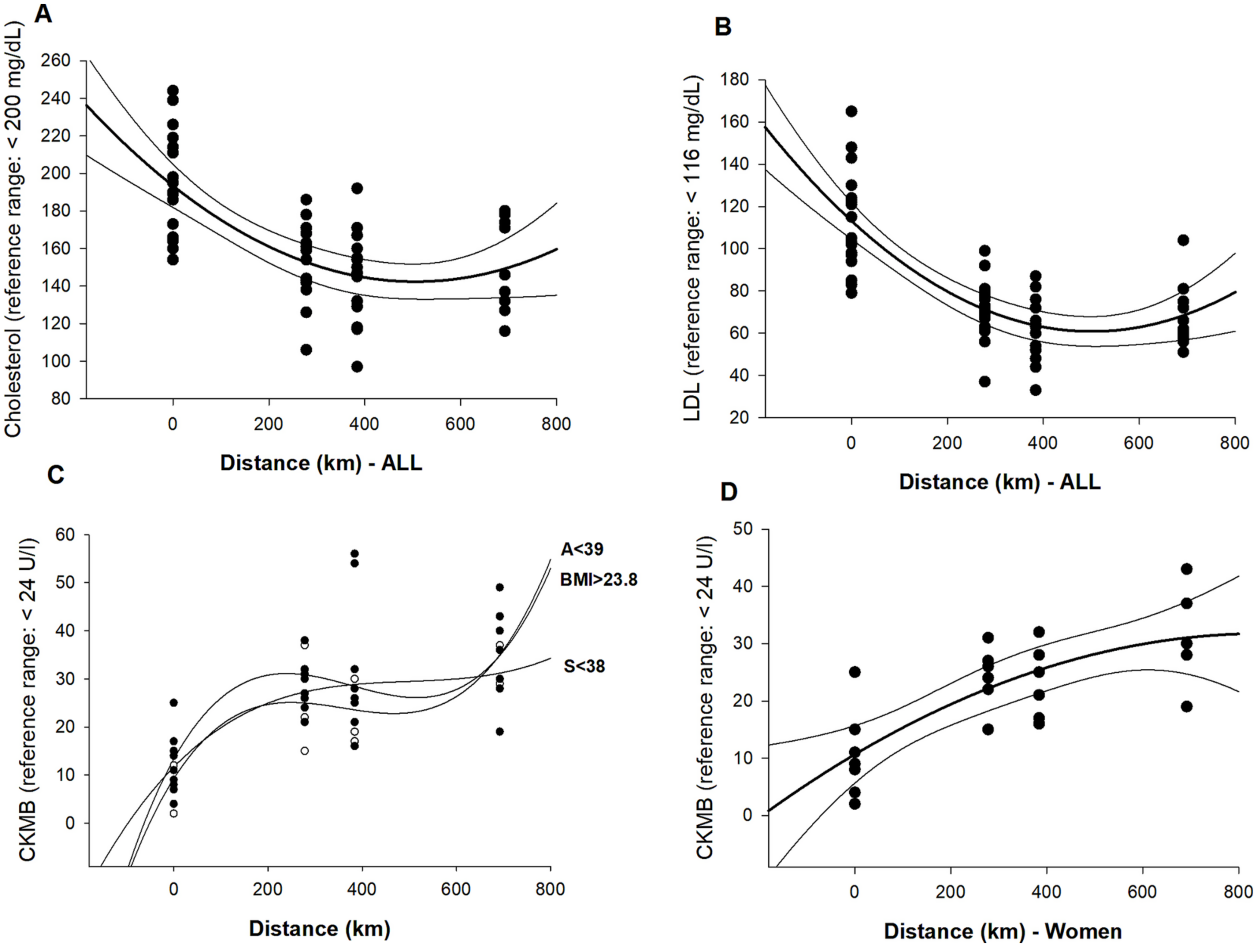
Graphs represent significant polynomial regression analyses of blood parameters over distance. **(A)** Cholesterol (*R*^2^ = 0.43). **(B)** LDL (*R*^2^ = 0.58), **(C)** correlations for CK-MB with AGE<39 (*R*^2^ = 0.5), BMI > 23 (*R*^2^ = 0.7), and S < 38 (*R*^2^ = 0.5), and **(D)** CK-MB in women (*R*^2^ = 0.59).

The results are presented as a whole group (ALL). Subgroups were additionally selected to determine significant differences between groups. Groups were split into FIN and NON, as well as M and W. For BMI, age, and speed, median values were determined to categorize the group above or below the median (Figures 3, 4).

### Energy balance

Energy intake (EI) was measured using food protocols. Food type, number of packs, kilocalories per 100 g, and product size were noted by the athletes. They had no food restrictions. Additionally, a standardized meal was provided at each checkpoint. The amount of kilocalories was estimated using recipes provided by the cooks. These were additionally checked and approved by a professional dietician at the Charité University Hospital of Berlin.

Energy expenditure was determined using the “Sense Wear Pro 3 Armband” (BodyMedia, Pittsburgh, PA, United States). The device collects information on skin temperature, electrical conductivity, and acceleration to estimate energy expenditure. It is worn on the upper arm of the participant (25–27).

### Body composition

Body weight was measured using a calibrated scale (Seca, Hamburg, Germany). Additionally, bioelectrical impedance analysis (BIA) was used to measure body composition. The device used was “BIA101” (Akern, Florence, Italy) with tetrapolar hand-to-foot electrodes. FM and fat-free mass (FFM) were determined by using the fat-specific formula (women <30% and men <20%) (28). FM was calculated by subtracting FFM from total body mass.

FFM male participants (kg) = 9.33285 + 0.0006636 height (cm)^2^–0.02117 resistance (*Ω*) + 0.62854 weight (kg) − 0.1238 age (years).

FFM female participants (kg) = 10.43485 + 0.00064602 height (cm)^2^–0.01397 resistance (Ω) + 0.42087 weight (kg) (28).

All assessments were made after a sleeping period under standardized conditions (i.e., voided bladder and before breakfast).

### Blood analysis

Blood was drawn via the antecubital vein, using a serum, Monovette (Sarstedt, Monovette, Sarstedt GmbH, Nümbrecht, Germany). The sample was centrifuged and pipetted to be stored in ultra-low freezers. A certified laboratory was assigned to analyze the blood tests (Labor 28 GmbH, Berlin, Germany, accredited at the “DAkkS,” 218 Deutsche Akkreditierungsstelle GmbH). CRP was obtained by the enzyme-linked immunosorbent assay (ELISA) method, using antibodies to determine the values, performed by a qualified laboratory assistant at the Charité University of Berlin (ENZO Life Sciences GmbH - Lörrach, Germany).

### Statistical analysis

Single “T-Tests” were used to identify differences between two groups. “One-Way Repeated Measures ANOVA” was used to identify differences in the same group over several time points (PRE, D1, D2, and POST). “Normality Test” (“Shapiro Wilk”) was performed to check for normality. In case of non-normal distribution, non-parametric tests were performed (Mann–Whitney U-test). To isolate the group, or groups that differed from the others, arithmetic means were determined, and the “Holm–Sidak” method was used to test between-group effects. Statistical significance was set at an alpha level below 0.05. Correlations were identified using polynomial regression orders 1, 2, and 3. Only correlations with a factor *R*^2^ > 0.3 are reported. Statistical program Sigma Plot 12.3 (Systat Software Inc., San Jose, Canada) was used to execute calculations and visualize graphs and scatterplots. Values are presented as means (based on individual means and then averaged at the group level), including standard deviations (± SD), if not otherwise indicated. The effect size between groups is determined using “Hedges’g.”

## Results

### General

Mean temperatures ranged from −10° to −17 °C and dropped to a low of −42 °C (23).

### Anthropometric data

A total of 22 participants took part in this study (14 men, 8 women), with a mean age of 41 ± 7.9 years. A total of 14 participants reached the finish line (FIN), of whom 5 were women and 9 were men. The average speed was 2.7 ± 0.3 km per hour, including resting and sleeping time, average moving velocity 4.3 ± 0.4 km per hour, and distance covered per day 63.7 ± 6.3 km.

Athletes (FIN) weighed 73.0 ± 10.8 kg at PRE and lost 4.3 kg throughout the race. Their FM loss was 3.1 ± 0.9 kg, and FFM loss was 1.2 ± 1.6 kg (Table 1).

**Table 1:** Overview of anthropometrics.

Presented as means + SD			**PRE**		**D1**		**D2**		**POST**		***p*-value**
			Mean	SD	Mean	SD	Mean	SD	Mean	SD	
			kg		kg		kg		kg		
Body mass	ALL		73.3	12.3	72.2	11.5	72.2	11.2	68.7	9.7	<0.001
	FIN		73.0	10.8	72.0	9.7	72.1	10.3	68.7	9.7	<0.001
	ALL	♂	80.5	7.9	78.6	9.7	77.0	8.7	73.2	6.5	
	FIN		78.2	7.6	76.2	7.0	76.2	7.3	73.2	6.5	
	ALL	♀	60.8	8.3	61.0	8.5	61.5	9.5	60.5	9.3	
	FIN		63.7	9.3	64.3	9.3	62.8	10.2	60.5	9.3	
FFM	ALL		58.9	11.0	59.2	10.4	60.2	10.0	56.9	8.7	<0.001
	FIN		58.2	13.2	58.5	9.0	58.4	9.3	56.9	8.7	<0.002
		♂	66.6	6.0	66.3	6.0	65.9	6.3	62.4	4.8	
		♀	46.9	4.2	48.0	4.3	48.8	5.0	47.9	5.1	
FM	ALL		14.1	3.3	12.7	3.3	13.0	3.3	11.7	3.3	<0.001
	FIN		14.8	2.4	13.4	3.6	13.1	3.4	11.7	3.3	<0.001
		♂	13.9	2.6	12.2	2.3	12.1	2.5	10.8	2.2	
		♀	14.4	4.2	13.5	4.3	14.8	3.9	13.3	4.0	
BMI	ALL		23.9	3.1	23.6	2.8	23.5	2.5	22.5	2.4	<0.001
	FIN		24.3	3.6	23.6	2.6	23.5	2.7	22.5	2.4	<0.001
		♂	25.2	2.4	24.4	2.4	24.1	2.5	23.1	2.4	
		♀	21.7	2.8	22.0	2.7	22.5	2.2	21.6	2.3	

### Energy balance

Energy Intake: A total of 53,049 ± 10,475 kcal was consumed in FIN (4,840 ± 819 kcal/ day). In NON, athletes consumed on average 4,768 ± 1,371 kcal/day.

Energy Expenditure: Total energy expenditure in FIN was on average 71,539 ± 10,585 kcal (6,628 ± 1,019 kcal/day). In NON, athletes expended on average 6,638 ± 1,235 kcal/day. At the beginning of the race, EE was higher compared to the end (day 2: 7,223 ± 1,855 kcal; day 10: 5,905 ± 1,813 kcal).

There was no statistical difference between FIN and NON.

Energy Deficit (ED): Participants´ EE was much greater compared to their EI. Thus, a significant ED resulted. In FIN, an ED of 18,442 ± 13,914 kcal (1,739 ± 1,343 kcal/day) was determined, in NON, 2,096 ± 1,452 kcal/day (Table 2).

**Table 2:** Overview of significant changes energy balance.

(a) Energy Intake—FIN				
Presented as means	TTL kcal	SD	kcal/ d	SD
**ALL**	**53,049**	10,474	**4,840**	819
**♀**	51,694	4,516	4,485	812
**♂**	53,802	13,601	5,038	1,237

(b) Energy expenditure—FIN				
Presented as means	TTL kcal	SD	kcal/ d	SD
**ALL**	**71,539**	10,585	**6,628**	1,018
**♀**	63,702	10,540	5,580	1,097
**♂**	75,022	9,976	7,094	925

(c) Energy deficit—FIN				
Presented as means	TTL kcal	SD	kcal/ d	SD
**ALL**	**18,442**	13,914	**1,739**	1,343
**♀**	12,193	13,254	1,026	1,077
**♂**	21,219	15,263	2,056	1,720

### Serum analysis

#### Cardiac parameters

Troponin T reached pathological levels in one athlete at D1, one at D2, and in two athletes at POST, with the highest values up to 20.5 ng/L. Older and faster athletes exhibited higher troponin T values at D1 and D2, as well as men at POST.

Almost all participants already had pathological values for NT-pro BNP and creatine kinase-myocardial band (CK-MB) at D1. NT-pro BNP declined again toward POST.

#### Stress parameters

CRP was elevated during the first part of the race and reached its highest level at D2 at 18.8 mg/dL. GOT, GPT, and CK showed increases, reaching pathological values at D1. A significantly higher serum level of CK was determined in faster runners at POST.

#### Lipids

LDL and cholesterol have declined significantly, especially during the first part of the race.

#### Thyroid parameters

Parameters fT3 and fT4 decreased significantly over race distance. Faster speeds showed significantly higher mean fT4 levels at D1 and D2. All values remained within the reference range.

### Correlations

Weak correlation: *R*^2^ = 0.1–0.39; moderate correlation: *R*^2^ = 0.4–0.69; strong correlation = *R*^2^ 0.7–0.89 (29).

#### ALL

Moderate and strong negative correlations for lipids over race distance except for HDL and triglycerides were detected (CHOL *p* = 0.001, *r*^2^ = 0.43; LDL *p* = 0.001, *r*^2^ = 0.58; non-HDL *p* = 0.001, *r*^2^ = 0.51).

#### FIN vs. NON

No inflammation markers correlated with distance among FIN, but, on the contrary, for NON, almost all correlated moderately with high significance (CRP *p* = 0.001, *r*^2^ = 0.47; GOT *p* = 0.001, *r*^2^ = 0.55; GPT *p* = 0.001, *r*^2^ = 0.50; CK *p* = 0.005, *r*^2^ = 0.52).

#### M vs. W

In women, NT-pro BNP and CK-MB correlated moderately. (NT-pro BNP *p* = 0.024, *r*^2^ = 0.45; CK-MB *p* = 0.003, *r*^2^ = 0.59).

#### AGE

AGE>39 showed a moderate correlation for NT-pro BNP; in contrast, AGE<39 for CK-MB. (NT-pro BNP *p* = 0.001, *r*^2^ = 0.48; CK-MB *p* = 0.001, *r*^2^ = 0.53).

#### BMI

For BMI > 23, almost all inflammation markers are correlated, as well as CK-MB. (CRP *p* = 0.011, *r*^2^ = 0.47; GOT *p* = 0.01, *r*^2^ = 0.48; CK *p* = 0.009, *r*^2^ = 0.48; CK-MB *p* = 0.001, *r*^2^ = 0.7).

#### Speed

Faster participants showed a moderate correlation for troponin T; on the contrary, slower participants showed a correlation for CK-MB. (troponin T *p* = 0.013, *r*^2^ = 0.47; CK-MB *p* = 0.007, *r*^2^ = 0.51).

## Discussion

This study was conducted to present a comprehensive overview of changes in body composition, metabolism, and several serum parameters in athletes. The YAU is a multistage race with short but optional recovery periods in between checkpoints.

### Estimated energy balance

A great energy deficit has been detected among the athletes (1,739 ± 1,343 kcal/day in FIN) studied in the YAU race. This was subsequently linked to body weight decrease, which is a common finding in ultramarathons (30–32). Whether this refers to a loss of fat mass only or lean mass also is described controversially in different studies (6, 22, 33, 34). Contrary to previous findings, a loss in lean mass has been determined in the YAU throughout all 4 years (PRE 58.9 vs. POST 56.6) (6, 22). However, this loss in lean body mass is quite modest, considering its large proportion in body composition.

A loss of FM with a mean of 2.4 kg was detected, yet no significant differences were found between the subgroups. Interestingly, a loss of body mass does not necessarily impair performance (35), which concurs with our findings. The winner of the race in 2017 lost 4.73 kg of body mass, and in FIN, athletes even lost up to 7.9 kg of body mass.

Energy intake (EI) varies greatly between participants (4,400 up to 6,600 kcal/day). Other comparable studies identified diverging values as well, varying from 3,303 to 7,156 kcal/day, albeit all in different ultramarathon settings (26, 36, 37).

In YAU athletes, a mean energy expenditure (EE) of 6,628 kcal/day was determined in FIN. Similar high values were identified in comparable studies, varying from 6,300 kcal/day to 7,400 kcal/day (26, 33, 38).

No relevant significant differences were detected in the subgroups. There was a difference in energy deficit (ED) with 1,739 kcal/day in FIN and 2,096 kcal/day in NON; however, it is not significant. This is most likely explained by the small sample size. It has been described that the energy deficit is greater in non-finishers (35, 39). ED is correlated with performance, but ultra-endurance athletes showed an EI between 36 and 54% of their EE (40), fewer than YAU athletes with 73% (FIN). The winner of a 402 km trail run in Arizona met 53% of their energy demand (41).

However, trying to reach the highest levels of energy intake is not the solution, as a large energy intake may lead to gastrointestinal stress (12, 36, 37). A few participants reported difficulties in this regard.

### Cardiac parameters

Troponin T is a highly sensitive marker of myocardial damage (42). Endurance exercise-related troponin T increase is a phenomenon that has been described thoroughly in the literature (3, 43, 44). During the YAU, troponin T increased significantly throughout the race, but the mean values were not pathological (<14 ng/L), except for one athlete at D1 and D2 and two athletes at POST, reaching a level of 20.5 ng/L. Interestingly, there was a significant difference in troponin T at POST between men with 11.2 ng/L and women with 4.3 ng/L. A meta-analysis on this subject described no clear differences for sex and age; however, exercise duration may have an influence (43). In addition, the YAU athletes who moved at greater speed had significantly higher troponin T elevations at POST, compared to slower athletes (S < 38 with 3.6 ng/L and S > 38 with 11.9 ng/L), indicating that intensity exerts an influence even in ultraendurance races with a comparably slow running speed.

Currently, the most well-received theory to explain the mechanism of troponin T release is the increased membrane permeability of cardiomyocytes (44). The increase was found to be correlated with post-race diastolic dysfunction and is inversely correlated with training mileage (43, 45, 46). Yet, the pattern of release is different from that observed in acute coronary syndrome and is likely physiological rather than pathological (47, 48), where the ischemic myocardiocyte injury does not progress to complete cell necrosis (43).

NT-pro BNP is a preliminary stage of BNP, which is a peptide predominantly released from the heart in response to ventricular wall stress (49). During the YAU, NT-pro BNP values were inclined to pathological levels (reference range: <125 pg./mL) and returned to reference values at POST only in a few athletes. Highest mean levels were reached at D1 (376.4 pg./mL), albeit the track being more challenging toward the end of the race due to the increased altitude. It can be speculated that the decline may constitute a form of adaptation process. A 60 k ultramarathon showed a 1.5-fold BNP elevation at POST and a 4,486 k multi-stage ultramarathon had a 3.5-fold elevation after 2,000 k (3, 50). Interestingly, there was a correlation for NT-pro BNP in older athletes (A > 38) and slower athletes (S < 38) over race distance; however, the latter had a correlation factor <4 (*p* = 0.049; *r*^2^ = 0.35). Yet, an indication that fitness level, sex and age relevant in NT-pro BNP release. A correlation with training status and endurance duration for NT-pro BNP values has also been described by other authors (20, 51, 52).

### Stress and inflammation parameters

Prolonged endurance exercise may have a negative effect on the immune system, leading to inflammation (9).

CRP was elevated up to 15.4 mg/L at D1. In a clinical setting, this would be considered pathological, although not severe (53). Contrarily, Belli et al. showed a 243-fold CRP increase during a 217 km mountain ultramarathon (54). CK showed significant differences, which are shown in Tables 3, 4. Mean values were pathological at all stages, with the highest levels at D1 (1,233 U/L ± 1,736). A higher serum level was detected in faster compared to slower racers (899 U/L vs. 482 U/L) at POST. We tested for correlation between FFM and CK rise, since it can be assumed that a CK rise is linked to muscle mass, yet with no significant results. A 24-h winter mountain running race in the Czech Republic, in freezing conditions, presented even higher results with CK elevations up to 7,000 U/L (32). Several other authors described a CRP and CK increase during and after marathons and ultramarathons (55–57) as an indicator of muscle damage and an inflammatory response.

**Table 3:** Overview of significant changes in blood parameters over distance.

Serum parameter		Reference range		Pre	D1	D2	POST	Significant change	*p*-values
CRP	C-reactive protein	< 0.5	Mean	1.7	15.4	18.8	3.7	PRE vs. D1	0.002
		mg/dL	SD	2.7	12.2	20.9	5.9	PRE vs. D2	0.003
GOT	Glutamat oxalacetat	< 35	Mean	26.6	100.6	86.7	68.9	PRE vs. D1	0.034
	Transaminase	U/L	SD	6.8	139.8	84.8	26.1		
GPT	Glutamat pyruvat	< 35	Mean	21.2	47.2	49.6	40.9	PRE vs. D1	0.013
	Transaminase	U/L	SD	9.9	42.3	44.1	21.1	PRE vs. D2	0.019
CHOL	Cholesterol	< 200	Mean	193.4	152.8	145.3	149.3	PRE vs. D1/D2/POST	all: < 0.001
		mg/dL	SD	26.6	20.6	24.0	22.8		
HDL	High density lipoprotein	> 40	Mean	63.9	70.9	69.6	70.0		
		mg/dL	SD	19.7	14.2	20.2	15.5		
LDL	Low density lipoprtein	< 116	Mean	113.2	71.5	63.4	68.5	PRE vs. D1/D2/POST	all: < 0.001
		mg/dL	SD	22.9	14.7	15.7	14.7		
Non-HDL		<130	Mean	127.2	82.3	75.7	79.4	PRE vs. D1/D2/POST	all: < 0.001
		mg/dL	SD	29.9	16.4	17.5	15.8		
TRIG	Triglycerides	< 200	Mean	79.6	54.6	62.6	54.1	PRE vs. D1	0.012
		mg/dL	SD	43.2	12.1	15.1	10.1		
TROP	Troponin T	< 14	Mean	4.9	6.5	5.7	7.8		
		ng/L	SD	5.1	5.4	4.3	5.3		
NT-pro BNP	n-terminal prohormone of	< 125	Mean	59.5	376.4	383.4	235.3	PRE vs. D1	< 0.001
	Brain natriuretic peptide	pg/mL	SD	50.0	377.4	333.1	337.5	PRE vs. D2	< 0.001
CK	Creatine kinase	< 140	Mean	118.6	1233.1	849.1	690.8	PRE vs. D1	0.007
		U/L	SD	50.3	1736.7	798.9	296.8		
CKMB	Creatine kinase MB	< 24	Mean	11.6	43.0	38.6	38.6	PRE vs. D1	0.027
		U/L	SD	5.5	56.9	37.3	14.6		
TSH	Thyroid stimulating hormone	0.27–4.2	Mean	2.1	2.0	2.3	1.6		
		mU/L	SD	1,3	1.4	1.3	0.8		
fT3	Triiodothyronine	2.0–4.4	Mean	3.5	3.2	3.1	2.5	PRE vs. POST	0.013
		pg/mL	SD	0.5	0.7	0.8	0.6		
fT4	Thyroxine	9.3–17	Mean	13.2	12.7	12.2	11.4	PRE vs. POST	0.013
		pg/mL	SD	1.9	1.4	1.3	1.9		

SD, standard deviation.

**Table 4:** Overview of significant changes in blood parameters in subgroups.

Category	Variable	Reference range	Checkpoint	Subgroup	Value	Subgroup	Value	Diff means	*p*-values	Hedges G
Cardiac parameters	TROP		D1	AGE<39	3.4	AGE>39	9.6	6.2	0.015	1.38
		< 14 pg./mL	D2	AGE<39	3.6	AGE>39	9.2	5.5	0.009	1.44
			POST	♂	11.2	♀	4.3	8.2	0.019	1.44
			POST	S > 38	11.9	S < 38	3.6	8.2	0.008	2.17
Inflammation	CK		POST	S > 38	899	S < 38	482	417	0.023	1.77
		<140 U/L	PRE	♂	141	♀	89	52	0.027	1.15
			POST	♂	944	♀	437	506	0.002	2.88
	GOT
<35 U/L	POST	S > 38	84	S < 38	53.2	30.2	0.04	1.25
Lipids	CHOL	< 200 mg/dL	D2	BMI < 23	154	BMI > 23	133	21	0.045	0.93
			D1	AGE<39	138	AGE>39	166	27.8	0.004	1.67
	HDL	> 40 mg/dL	D1	AGE<39	67	AGE>39	73	6.4	0.002	2.02
			FIN	♂	59.8	♀	80.2	20.4	0.039	1.47
	LDL	< 116 mg/dL	POST	BMI < 23	75.6	BMI > 23	60.6	15	0.046	1.24
Thyroid	fT4	9.3–17 pg./mL	D1	S > 38	13.3	S < 38	11.9	1.4	0.04	1.08
			D2	S > 38	13	S < 38	11.4	1.5	0.038	1.29

CK and CRP levels depend on multiple factors, including sex, age, ethnicity, baseline, muscle composition, and speed (55, 58, 59). CK is also correlated with exercise duration (18) and may be influenced by cold temperature. Teleglow et al. showed an increase of approximately 8.5% in temperature under Arctic conditions (60). During the YAU, the values remained below the reference for rhabdomyolysis (< 1,500 U/L) except for two athletes. One of them even reached 7,325 U/L at D1. This athlete suffered from gastrointestinal distress and loss of appetite; however, he was the fastest athlete and the winner of the race in 2017.

GPT is a specific liver injury marker, and GOT is found in liver cells and muscle cells (9, 18). GOT and GPT both show increases in the first part of the YAU race. GOT between PRE and D1 (difference in means: 76.6 U/L) and GPT between PRE and D1 as well as D2 (difference in means: 28.3 U/L and 26.4 U/L), with mean pathological values for GOT from D1 onward. Furthermore, faster speed resulted in a significantly higher enzyme release for GOT at POST, compared to slower athletes (S > 38: 84.0 U/L vs. S < 38: 53.8 U/L). A 768-km multi-stage ultra-trail case study also showed an elevation in GOT at POST of 167% and GPT of 159% (56). It is assumed that the increases were due to muscle enzyme release rather than liver damage (17). Especially, the release of muscular GOT can occur with exercise (61).

In the YAU, there was a remarkably significant correlation in NON for all inflammation markers (CRP, GOT, GPT, and CK) with race distance, but none for FIN. Thus, elevated inflammation markers could be a first sign of unsuccessful performance (Table 5).

**Table 5:** Overview of significant moderate and strong correlations over distance.

		CRP	GOT	GPT	CK	CHOL	LDL	NON-HDL	TROP	NT-pro BNP	CKMB	fT4
ALL	Order*					2	2	2				
	*p*-value					0.001	0.001	0.001				
	Correlation factor					0.433	0.586	0.518				
FIN	Order*						2	2				
	*p*-value						0.001	0.001				
	Correlation factor						0.515	0.742				
NON	Order*	1	1	1	1	1	1	1			1	1
	*p*-value	0.001	0.001	0.001	0.005	0.001	0.001	0.001			0.003	0.032
	Correlation factor	0.479	0.553	0.504	0.406	0.52	0.754	0.505			0.438	0.458
♂	Order*					2	2	2				
	*p*-value					0.002	0.001	0.001				
	Correlation factor					0.457	0.585	0.496				
♀	Order*		3		3	2	2	2		3	3	
	*p*-value		0.003		0.028	0.005	0.001	0.001		0.024	0.003	
	Correlation factor		0.415		0.431	0.461	0.634	0.619		0.455	0.598	
AGE>39	Order*					2	2	2		2		
	*p*-value					0.001	0.001	0.001		0.001		
	Correlation factor					0.433	0.667	0.551		0.483		
AGE<39	Order*					2	2	2			3	
	*p*-value					0.001	0.001	0.001			0.001	
	Correlation factor					0.491	0.584	0.509			0.531	
BMI<23	Order*					2	2	2				
	*p*-value					0.001	0.001	0.001				
	Correlation factor					0.538	0.674	0.576				
BMI>23	Order*	3	3		3		2	2			3	
	*p*-value	0.011	0.01		0.009		0.001	0.001			0.001	
	Correlation factor	0.477	0.484		0.489		0.527	0.509			0.7	
S > 38	Order*					2	2	2	3			
	*p*-value					0.002	0.001	0.001	0.013			
	Correlation factor					0.475	0.621	0.642	0.475			
S < 38	Order*					2	2	2			3	
	*p*-value					0.004	0.001	0.005			0.007	
	Correlation factor					0.44	0.578	0.441			0.517	

Correlations are only stated with a correlation factor *R*^2^ > 0.4.

### Lipids

Lipids are an important source of energy during exercise, especially during low-intensity and moderate-intensity exercises (11). The effect of cold environments on substrate metabolism during prolonged exercise, however, is less certain (62). In our study, triglycerides, cholesterol, LDL, and non-HDL decreased significantly over the race course, with the greatest change at D1 (277 km) compared to PRE. Triglycerides decreased on average 31%, cholesterol by 21%, LDL by 36%, and non-HDL by 35%. Interestingly, a slight increase at checkpoint D2 was identified for triglycerides. Similarly, a reduction of over 50% for triglycerides (133 vs. 64 mg/dL) was noted during an ultramarathon of 133 km length in Brazil (19), and a 1,600 km foot race in Australia showed increasing levels after day 4 (63). This finding is consistent with our results, even though the race length was more than 2-fold.

Remarkably, lipids correlated over race distance in all participants and therefore in all subgroups (age, BMI, sex, speed as well as FIN and NON).

Fatty acids produced from thyroid hormone-induced lipolysis are substrates for increased thermogenesis (64), which is a core aspect in the YAU due to the Arctic temperatures. Brown adipose tissue may accelerate the serum clearance of triglycerides (65) as well as induce a reduction in serum cholesterol and LDL (66). Triglycerides are also strongly influenced by the consumption of food and beverages (67). Additionally, due to the female sex hormone beta estradiol, women have increased lipid and decreased carbohydrate use during endurance exercise, compared to men (68). Interestingly, women showed higher HDL levels at Post compared to men (M:59.8 mg/dL vs W: 80.2 mg/dL).

In HDL, changes are usually not seen during the race (19) but in some cases, after the race has finished (33, 69). In the YAU, a trend toward increasing values was noted. Lipid transfer to HDL is higher in marathon runners than in sedentary individuals, but the marathon itself may acutely inhibit lipid transfer (70). HDL is also influenced and increases at cold Arctic temperatures (60). Substantial changes in lipid values were seen for all participants, which may have resulted in a significant loss of FM.

### Thyroid hormones

There is very little literature on thyroid hormones while running an ultramarathon in the cold. It is known that thyroid hormones play a major role in cold adaptation (71, 72), by inducing most of the non-shivering thermogenesis in brown adipose tissue (72, 73). Whether or not this influenced YAU participants is not clear. Several authors describe that a fT3 plasma decrease is induced when exposed to the cold (71, 74, 75). Living under Arctic conditions can induce “polar T3 syndrome,” a condition associated with decreased fT3 levels (76). In our study, the data acquired might underlie fluctuations due to the circadian rhythm (77). However, fT3 and fT4 decreased significantly over race distances but stayed within reference levels. There were no significant changes in TSH. Furthermore, physical exercise is associated with a reduction in pituitary–adrenal activation (78, 79). The loss of body fat and the increase in the amount of energy used during endurance exercise are processes largely regulated by thyroid hormones (80, 81). Previous studies indicated a link between weight loss and decreases in plasma fT3 levels, respectively, and mild thyroid dysfunction (82, 83). FIN men lost on average 4.9 ± 2.1 kg, and FIN women 3.2 ± 1.8 kg during the YAU.

In our previously published study by Rundfeldt et al., we could determine an overall decrease in psychological wellbeing, but FIN scored lower in the profile of mood states (POMS), exhibiting a better mood state (21). In this study, NON showed a correlation over distance with a fT4 decrease, indicating a possible link to impaired mood.

Furthermore, changes in thyroid hormones may negatively affect cardiac function (84), especially impaired diastolic function (85). This, in turn, may lead to increased concentrations of NT-pro BNP (86), as indicated by our results.

A “low T3-syndrome” or euthyroid sick syndrome is described in several human conditions. It occurs during fasting, diabetes, acute or chronic inflammatory, or malignant diseases, in the perioperative period, etc. (87, 88) and is described as a negative outcome (89–92). Lower limits vary by study. Guo et al. set baseline levels at 2.4 pg./mL (93). Mean values in our study were 2.5 pg./mL (± 0.6) at POST. However, the treatment of thyroid hormone abnormalities is as controversial as its physiological interpretation (94).

During the YAU, the data suggest that the decrease in fT3 and fT4 presumably interacted with thermogenesis, energy expenditure, as well as mood state, and further regulatory systems, and that it may have contributed to race drop out.

## Limitations

As with other field studies in extreme environments, only a small number of athletes compete in the Yukon Arctic Ultra: a total of 117 athletes were enrolled during the 4 investigated years. Of those, 19% (*n* = 22) participated in our study. The combination of race results was deemed necessary in order to increase the number of participants and thus statistical power.

In 2013, the investigation was conducted for the first time, to an extent as a feasibility study. Ever since 2015, blood markers have been evaluated throughout all the following years, although some blood markers were only evaluated in two different years, which may induce potential biases.

Regarding energy analysis, questionnaires were not filled out completely by every athlete. Food at checkpoints was standardized, and athletes received all the same serving sizes. However, these meals were estimated using recipes that have been checked by professional dieticians.

Some Sense Wear devices did not record the entire racing time. Thus, the recorded time was extrapolated using the manufacturer’s software.

## Conclusion

This field of research provides insight into serum changes and energy balance during ultra-endurance racing under Arctic conditions. Athletes should be aware of their great energy demand and strive to reduce energy deficit to a minimum. Substantial serum alterations can be indicative of race performance. Inflammation parameters correlate with dropout rates and could therefore be used as a prognostic marker. Lipids dropped impressively, influenced by both exercise as well as the Arctic temperatures. The fT3 decrease can be associated with a “polar-” or “Low T3- syndrome” with its multiple influences on psychological and physiological implications. Whether these alterations are pathological or without clinical relevance is still subject to research. At this point, it can be suggested that athletes try to monitor alterations during training and competition. The results should be incorporated into planning and training and should be highly individualized to each athlete’s requirements and needs.

## Data Availability

The raw data supporting the conclusions of this article will be made available by the authors, without undue reservation.

## Glossary

D1: first checkpoint during the race at 277 km
D2: second checkpoint during the race at 383 km
EI: energy intake
EE: energy expenditure
ED: energy deficit
FFM: fat-free mass
FM: fat mass
PRE: checkpoint at the starting point at 0 km
POST: checkpoint at the end of the race after 690 km
SD: standard deviation
TTL: total
YAU: Yukon Arctic Ultra
ALL: all athletes (FIN + NON)
FIN: finisher
NON: non-finisher
AGE<39: age < = 39 years
AGE>39: age > 39 years
BMI < 23: BMI < 23.8 kg/m^2^
BMI > 23: BMI > 23.8 kg/m^2^
S < 38: speed (race distance) < 38.8 km/day
S > 38: speed (race distance) > 38.8 km/day
M: men
W: women
